# How the 26S Proteasome Degrades Ubiquitinated Proteins in the Cell

**DOI:** 10.3390/biom9090395

**Published:** 2019-08-22

**Authors:** Bernat Coll-Martínez, Bernat Crosas

**Affiliations:** 1 Department of Cell Biology, Institute of Molecular Biology of Barcelona (IBMB), Consejo Superior de investigaciones Científicas (CSIC), Baldiri i Reixac 4-10, 08028 Barcelona, Spain; 2 Institut Químic de Sarrià (IQS), School of Engineering, Universitat Ramon Llull, Via Augusta 390, 08017 Barcelona, Spain

**Keywords:** ubiquitin, proteasome, ATPase motor, protein degradation mechanism

## Abstract

The 26S proteasome is the central element of proteostasis regulation in eukaryotic cells, it is required for the degradation of protein factors in multiple cellular pathways and it plays a fundamental role in cell stability. The main aspects of proteasome mediated protein degradation have been highly (but not totally) described during three decades of intense cellular, molecular, structural and chemical biology research and tool development. Contributions accumulated within this time lapse allow researchers today to go beyond classical partial views of the pathway, and start generating almost complete views of how the proteasome acts inside the cell. These views have been recently reinforced by cryo-electron microscopy and mechanistic works that provide from landscapes of proteasomal populations distributed in distinct intracellular contexts, to detailed shots of each step of the process of degradation of a given substrate, of the factors that regulate it, and precise measurements of the speed of degradation. Here, we present an updated digest of the most recent developments that significantly contribute in our understanding of how the 26S proteasome degrades hundreds of ubiquitinated substrates in multiple intracellular environments.

## 1. The Proteasome in Its Challenging Habitat

The 26S proteasome has a structural configuration that confines the proteolytic active sites in a location unreachable for native and functional proteins, thus preventing uncontrolled degradation. The proteolytic active sites are found in the interior of a barrel-shaped core particle (CP or 20S). The entrances of the tunnel, placed at the distal ends of the barrel, are commonly occupied by the regulatory particle (RP or 19S), a sophisticated protein assembly that acts as a substrate processing machine [1]. As described in more detail in this text, the regulatory particle has the important role of receiving, deubiquitinating, unfolding and translocating substrates to the CP and it adopts different configurations depending on the activity states they exhibit [2]. Moreover, conformationally distinct proteasomes may show different subcellular distributions depending on functional requirements in each cellular type and environmental situations, as discussed below.

**Figure 1:**
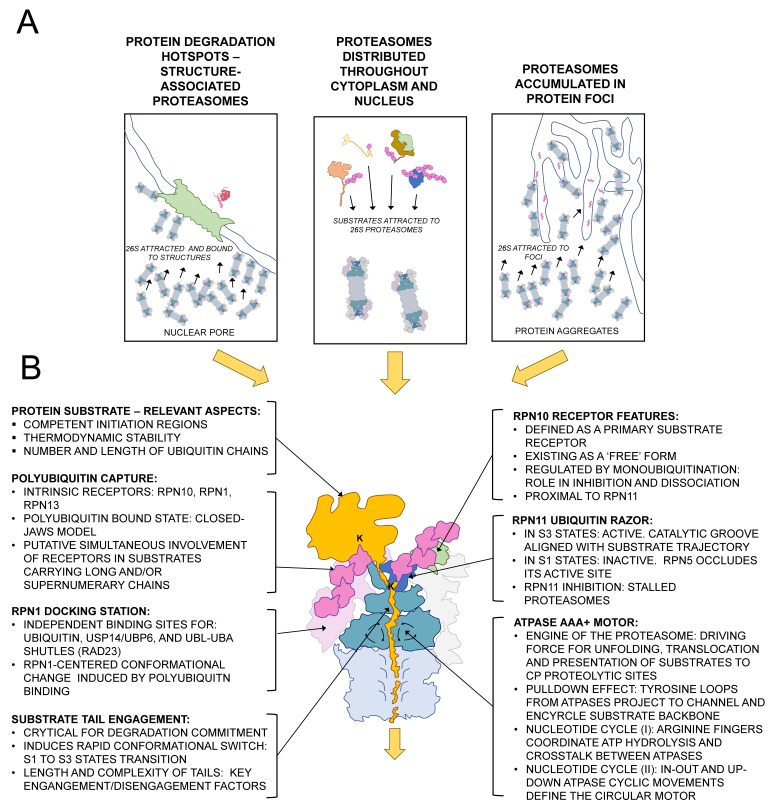
Schematic representation of the main aspects in 26S proteasome mechanism. (**A**) Different possible proteasome environments inside cells are presented, focusing on the works commented in the text. (**B**) Relevant points in the mechanism of protein degradation by the 26S proteasome.

Proteasomes are distributed throughout the cell, detected in the cytoplasm and in the nucleus, and they show hotspots in distinct intracellular regions or specific sites with high protein metabolism or with specific protein degradation requirements (Figure 1A). Abundant pictures of the landscape of proteasome distribution inside distinct cell types are not yet available, but there are evidences that indicate that proteasomes, in addition to a scattered distribution inside the cell, may be attracted towards multiple cellular sites in their effort to interact with protein substrate pools targeted for degradation or accumulated because of the difficulty in degrading them. For this topic, several labs leading whole-cell tomography and high resolution cryo-electron microscopy technologies have made notable contributions. Namely, Baumeister lab has provided remarkable insights in conformation and localization of proteasomes inside cells. A nice example of that, as reported by Guo et al. [3], is the observation in neurons of strong recruitment of proteasomes into poly-Glycine-Alanine (poly-GA) aggregates, a type of protein aggregates generated and observed in amyotrophic lateral sclerosis (ALS) and frontotemporal dementia (FTD). These two diseases have in common a severe alteration of the function of the ubiquitin-proteasome system, a trait exhibited by multiple neurodegenerative disorders (Alzheimer, Parkinson, for example). It is important to highlight that although the molecular mechanisms underlying neuronal dysfunction are not well understood, there is a remarkable amount of data showing that the ubiquitin-proteasome system (UPS) is a pivotal actor in this type of disorders. The most common alteration linked to ALS and FTD is a mutation in the *C9orf72* gene, consisting in a massive expansion (thousands of copies) of a G_4_C_2_ repeat in a non-coding region. It is still unclear how this mutation mediates neural toxicity, with several plausible options: (i) the aberrant RNAs containing repeats show severely decreased translation, affecting the function of the produced protein, (ii) aberrant RNA abnormally interacts with other cell components or (iii) repeat-associated non-ATG (RAN) translation of the expanded noncoding region generates toxic products [4]. The third option was validated by the detection of all six combinatorial possible products of sense and antisense unconventional translation products in aggregates from brains of ALS/FTD patients [5]. Among the six G_4_C_2_ repeat translational possible products, the most abundant is the one generating poly-GA repeats. It has been shown that the expression of poly-GA produces toxicity and accumulates UPS factors. In a remarkable contribution, Fernández-Busnadiego group shed light in the structural configuration in poly-GA aggregates and in the recruitment of proteasomes in those aggregates in their attempt to clear aberrant proteins. They analyzed proteasomes-aggregates interaction in neurons, the naturally occurring environment [3]. First, they observed that poly-GA forms amyloid-like ribbons in neurons, which show bifurcated and polymorphic fibers. Second, they verified that the interior of the poly-GA inclusions was populated by a high number of 26S proteasomes, accompanied by a less abundant pool of TRiC/CCT chaperonin. Moreover, an important concentration effect of proteasomes in the aggregate, quantified as a 40-fold increase with respect to the rest of cell body or to control cells, was observed. Since a significant global increase of proteasome particles was not observed in aggregate-containing cells, they concluded that the formation of the poly-GA body causes a sequestration of proteasomes, which are removed from other cellular loci where they certainly carry out other tasks in a normal context. The numerous population of proteasomes within poly-GA aggregates enabled the authors to perform reliable proteasome conformational analysis. They observed that 76% of proteasomes associated to poly-GA were doubly capped (RP-CP-RP full size assembly) proteasomes. This suggests an effect of stabilization of the CP-RP interaction within poly-GA aggregates. This is remarkable since previous studies suggest that the most abundant form of proteasome in the cell, which accounts for a 73% of the pool, is singly capped (RP-CP) [6]. The sorting analysis of the referred work provided valuable information on the activation status of the proteasomes found in the aggregates. They observed close to 40% of proteasomes in a substrate processing conformation (S2–S4 configurations), a ratio higher than that found in normal neurons. They could observe 14% of proteasomes adopting a substrate-commitment state (S2-like), and 23% adopting an S4-like conformation, meaning actively translocating proteasomes, which is considered a highly transient conformation. These complexes showed a prominent density volume in the substrate interacting region, indicative of the presence of an engaged substrate (and maybe additional cofactors, such as shuttling factors or enzymatic machinery). Altogether, this suggests that S4-like substrate-engaged proteasomes are stalled proteasomes, and they represent the most abundant sub-configuration pool in the aggregates. It is important to emphasize that this is the first work in which bona fide assignation of conformational states (S1 and S2–S4 configurations) was performed out of cell tomograms. When the variables ‘proteasome state’ and ‘distance of proteasomes from the poly-GA ribbon’ were analyzed in tomograms, an interesting correlation was found. S4-like proteasomes were enriched in the pool of proteasomes contacting the aggregates, and S2-like proteasomes were enriched in pools of proteasome showing no contact with aggregates. These suggests that physical association with poly-GA aggregates affects the functional state of the proteasome, perhaps due to the incapacity of proteasome to rapidly degrade the aberrant protein. This work represents a good example of structural and functional analysis of the proteasome in its habitat. An additional relevant work was provided by Albert et al. [7]. In this work, *Chlamydomonas Reinhardtii* algae cells were analyzed by tomography. Chlamydomonas, despite being evolutionarily highly distant from higher pluricellular eukaryotes, shows a strictly conserved ubiquitin sequence and contains all proteasome proteins [8]. In addition, it is an excellent cellular model for whole-cell cryo-EM due to its poorly crowded proteome. Using this model, and after considerable effort in tomogram acquisition and analysis, the authors could establish the RP-CP interaction status (double-capped, single-capped and free forms of proteasome sub-particles) and the exact localization of these forms. An important observation was that, whereas proteasomes showed a scattered distribution along the cytoplasm and the nucleus, they concentrated in high number in the inner nuclear membrane and in the nuclear pore complex areas. They estimated concentrations of proteasome particles in each region and they observed that, while cytoplasm and nucleoplasm showed concentrations around 150 nM, in the inner membrane proximity areas, it reached up to 8.11 µM. When a detailed inspection of particle status was performed, it was observed that RPs, in addition to S1 (substrate free) and S3 (substrate processing) states, showed membrane-tethered and nuclear pore basket-tethered assemblies (in addition to “free”, unbound, status). Rigid-body fitting of refined tomogram averages with high-resolution 26S structures revealed that the interaction of proteasomes with those structures is mediated by Rpn9, a lid subunit with no attributed interactive roles, other than being part of the subcomplex and contributing to Rpn10 docking. Interestingly, all basket-tethered and membrane-tethered proteasomes localized in the nuclear side and represented 43% of the nuclear proteasome population. These associated proteasomes were detected in hotspot regions defined by the nuclear pore complex and environs. Authors suggest that these bound proteasomes define two functionally distinct population groups. The first one, the basket-tethered group, shows the optimal position to recruit soluble proteins transiting the central channel of the nuclear pore, while the second one, including membrane-tethered proteasomes, could interact with membrane proteins traveling through peripheral channels. Altogether, this proteasome crowd could represent a checkpoint of quality control of proteins crossing this important intracellular border. This type of study will certainly proliferate during next years, as methodology gets more accessible, and panoramic views of proteasomes populations will reveal significant proteasome distribution/state/function relationships, and maybe these will be correlated with protein substrate pools involved in each cellular context.

## 2. Detailed Kinetics of Substrate Processing, Translocation and Degradation

Regardless of the intracellular site of a given proteasome particle, its anchoring status and the associated factors that participate in substrate recruitment, the proteasome exhibits a kinetic mechanism that makes possible the continuous processing and proteolysis of a massive flow of substrates that have to be cleared from the cell in order to avoid their accumulation. Whichever is the metabolic and functional status of a cell, whichever are the stress inputs that act in a cell, the task of timely degradation of the intracellular protein pool is highly challenging. Key steps in the mechanism of protein degradation by the proteasome are discussed in the following sections, and summarized in Figure 1B.

As well-established in the literature, proteins are normally signalized to the proteasome by means of ubiquitin labels attached covalently to a lysine residue, usually in a chained form [9,10] The process of protein polyubiquitination is carried out by a highly specialized and diverse enzymatic system, which includes ubiquitin activating enzymes, ubiquitin conjugating enzymes and ubiquitin ligases [1,11]. In order to describe the molecular events that take place in the proteasome during degradation of polyubiquitinated proteins, Andreas Martin lab has applied high resolution Cryo-EM methods, in parallel with proteasome purification and recombinant production tools, protein polyubiquitination, specific amino acid labeling, Förster resonance energy transfer (FRET) and anisotropy assays. This way, Martin’s group has accomplished a fantastic series of publications, starting with Lander et al., 2012 [12], and ending with two of the most remarkable contributions, De la Peña et al., 2018 and Bard et al. 2019 [13,14], in which details of proteasome conformational status are linked to substrate processing kinetics and translocation.

To dissect proteasome substrate processivity and conformational changes they developed FRET-based assays sensitive to specific proteasome intrinsic events. To do that, the unnatural amino acid 4-azido-l-phenyalanine (AzF) was introduced in key positions of proteasomes subunits and substrates by means of amber codon incorporation system [15]. The presence of AzF makes possible the introduction of DBCO-linked fluorophores, to produce Cy3 and Cy5 donor-acceptor pairs, and then track fluorophores proximity induced by conformational changes or substrate-enzyme productive binding by FRET signal patterns. As an initial characterization, they monitored the conformational change that brings the 26S proteasome from a silent state (S1) to a substrate processing state (S3-like), which causes the rotation of the lid with respect to the base, and thus a 40 Angstroms shift in the distance between lid Rpn9 Ser111 and base Rpt5 Gln49. Therefore, these positions were mutated to AzF in separated lid and base purifying systems, and Cy3 and Cy5 where chemically linked to AzF residues. This way, in reconstituted proteasomes, the conformational change induced during proteasome activation could be measured as a reduction in the distance between Rpn9-S111Azf-Cy3 and Rpn5-Q49AzF-Cy5 which resulted in an increase of FRET signal. This approach could be used to scan those conditions that promote the adoption of activated conformations, linked to a RP rotation and substrate engage-like S3 state. Incubation of proteasomes with ATP was used as a reference value for activation, standardized as 100% of the FRET signal. In identical conditions, ATPγS (adenosine-5′-*O*-((3-thio)triphosphate) analog produced 130% of the signal. This ATP analog has been shown to induce substrate-engaged states, therefore, an increase in FRET signal could be expected, and in fact, was confirmed. The interaction of the proteasome with the deubiquitinating enzyme Ubp6 has been linked with protein degradation delay, thereby synchronizing the pace at which the proteasome subunits interact while processing ubiquitinated substrates [16,17]. The addition of tetra-ubiquitin and the catalytically inactive Ubp6 mutant (C118A mutant), which maintains the allosteric effects with the proteasome, caused an activation of 120% with respect to ATP alone. Moreover, the addition of an ubiquitinated substrate to the proteasome caused an increase of near 130% of the signal. This value was further increased to near-140% when an ubiquitinated substrate and o-phenanthroline (o-PA) were added together. This compound acts as an Rpn11 inhibitor, disabling its deubiquitinating role. This assay, a FRET assay in vitro, allowed to show that the proteasome adopts an RP-rotated S3 state when it degrades a substrate, and facilitated an experimental basis for tracking proteasome activation states during protein degradation reactions.

To go further on the characterization of the kinetics of protein processing by the proteasome during coordinated proteolysis, Bard et al. 2019 [14] developed fluorescence-based assays using multiple labeling strategies. First, they used as a model substrate containing a small folded domain of the giant muscle protein Titin, with an unstabilizing mutation (Titin-I27^V15P^) and an unstructured tail from Cyclin B, containing one single lysine residue (23-K-35). The presence of a defined unstructured tail (35 amino acids), encompassing a ubiquitination site (K23), ensured a constant docking geometry and a directional tail engagement and degradation. On top of that, two sites were defined for fluorescent labeling: (1) the N-terminal end of the folded domain (through Sortase-A labeling), which allowed anisotropy measurements to track the exact time required for protein degradation [18], and (2) a unique cysteine residue at a position flanking ubiquitination site, at the tail, which allowed measurements of tail engagement. They designed single-turnover experiments by using an excess ratio of proteasome in order to set up the optimal conditions of degradation and define degradation based on reliable initial velocities. They observed the following behavior in the anisotropy values: first, a rapid increase; second, a slower increase, both indicating sequential kinetics, and third, an exponential decay. This two-step increase was deconvoluted with additional assays. They performed the same assay with Rpn11AXA proteasomes, incapable to promote substrate deubiquitination in the absence of Ubp6, and observed only the first quick and short increase in anisotropy, but no additional changes were observed. When the same assay was carried out in the presence of ATPγS-bound wild-type proteasome, no increase or decay was observed at all. These observations suggested that the quick initial increase was ATP dependent, probably representing the process of tail engagement into the AAA+ motor. The second slower increase was deubiquitination-dependent and reported the process of ubiquitin removal and mechanical pulling of the substrate into the entrance of the motor. After that, the decay was observed, which includes the process of unfolding, translocation and degradation of the protein in the CP producing small peptides. The initial period of linearity of the decay was considered the correct readout of the degradation, since the second slower phase of the decay in anisotropy was attributed to the degradation of suboptimal forms (partially aggregated, poorly ubiquitinated substrate). With these considerations, the total degradation time for ubiquitinated Titin-I27^V15P^-23-K-35 was a time constant of 18,1 s, divided in 7 s of increase and 11 s of decay.

To proceed further in the dissection of this process, authors used again FRET measurement experiments. To track the kinetics of the process of tail insertion, they designed a FRET assays based on energy transfer from a donor fluorophore attached in the linker region between N-domain and ATPase domain of Rpt1 (by means of the Rpt1-I191AzF-Cy3), to the acceptor fluorophore placed at the insertion tail of the substrate (titin-I27V15P-23-K-35-Cy5). In this assay, proteasomes were pre-treated with o-PA, preventing further processing. Then, proteasome and substrate were mixed in a stopped-flow device coupled to a fluorimeter and signal was acquired. The measurements provided the data from proteasomes stalled in a tail-engaged state, exhibiting a high FRET signal due to the stabilized proximity between the ATPase-placed FRET donor and the substrate tail-placed FRET acceptor. This assay showed a quick increase in FRET signal, revealing a constant of 1.6 s for tail insertion. Next, the kinetics of the conformational change from S1 to S3 state was measured. To interpret the data, it was considered that the binding of polyubiquitin does not induce any conformational change, and that the first relevant event in that aspect is the productive insertion of the tail, which triggers, and thus it precedes, activation of the proteasome. To approach that, FRET signal from Rpn9-S111Azf-Cy3 to Rpn5-Q49AzF-Cy5, in o-PA treated proteasomes, was acquired. After a short decay in FRET signal, a quick increase of FRET signal was observed, with a constant for conformational change of 2.2 s. The quick and short decay of the signal caused a delay in the kinetics with respect tail insertion, and overlapping graphs showed that the exact delay was 0.4 s. That delay corroborates the notion that tail insertion is the first event, and that the conformational change is faster than the process of productive substrate tail insertion (0.6 vs. 1.6 s).

In the sequence of events, the next step is the attack to the isopeptide bond that attaches the substrate to polyubiquitin, step that is crucial to avoid stalling of the proteasomes in the middle of the degradation process and also to promote de recycling of the chained ubiquitin molecules attached to substrates. Another conceptual relevance of substrate deubiquitination by the proteasome is that it culminates a signaling process that involves a sophisticated group of enzymes, specificity factors and high amount of devoted energy. Therefore, an accurate control of this reaction is required in order to secure the efficiency of the whole process. To address this step in the same experimental framework, the authors designed a FRET-based assay that used a version of ubiquitinated Titin substrate in which a donor-labeled ubiquitin was proximal to an acceptor fluorophore, linked to the tail of the substrate. Thus, Titin ubiquitination generated a high-energy transfer status in this substrate, the decay of which would be tracked in the reaction of deubiquitination. This was achieved upon proteasome and substrate mixing in a stopped-flow device, and the time constant was measured as 6.8 s. In this case, given the sequence of events, total time for deubiquitination, would be a summation of the binding, tail insertion and deubiquitination.

**Figure 2:**
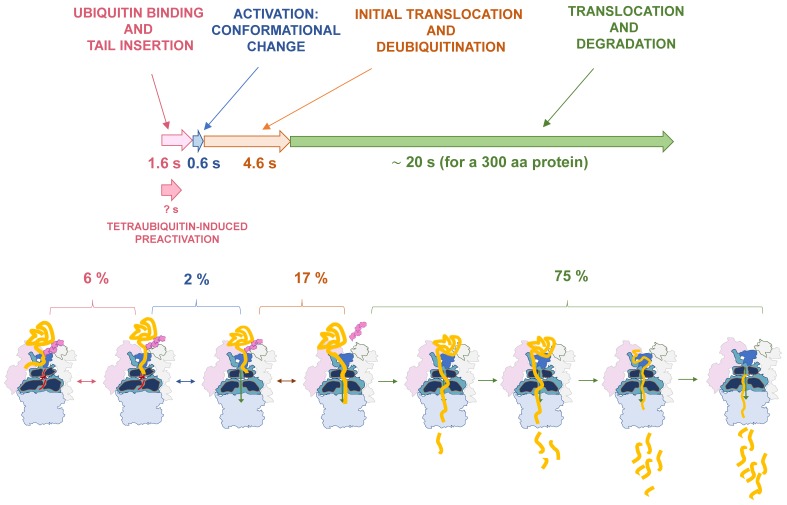
Dynamic representation of protein degradation by the proteasome. Steps described in the text are shown in different colors, on top, and the information related to each phase is represented in consistent color patterns. Arrows indicate times assigned for each step, based on Bard et al. 2019 [14], adapted to a hypothetical 300 amino acid proteins. The length of the arrows is proportional to estimated times. Percentage numbers indicate the fraction of the total processing time invested in each step. Cartoons representing each step are included below.

Therefore, after time dissection of the process, the total kinetics of degradation of Titin model substrate was established as: Tail insertion, 1.6 s; conformational change, 0.6 s, deubiquitination, 4.6 s, unfolding and proteolysis, 11.2 s. Further studies are required to determine whether this time dissection tends to be conserved among different substrates, other than Titin. In any case, this is the first report of the precise steep-by-step time dissection of the mechanism of protein degradation by the proteasome. Thus far, the speed of the proteasome in degrading a ubiquitinated substrate, based on Titin proteolysis, was estimated to be approximately 20 s per a 300-amino acid protein (Figure 2).

Interesting additional information concerns the parameters that influence the turnover of ubiquitinated substrates by the proteasome. Bard et al. [14] approached this important point using different versions of the model substrate, namely, with different number of ubiquitin chains, with distinct lengths and complexities of the initiation regions and with different intrinsic thermodynamic stability. They observed that there is a hierarchy of requirements that influence protein degradation (at least, this observation is valid for the model substrate used, in the specific conditions of this work). They determined that a competent initiation region is necessary for the commitment of the substrate, in agreement with previous works [17,19]. Thus, 25 to 35 amino acid long tails facilitate the engagement of the polypeptide into the AAA+ motor, promoting a rapid degradation. Shortening the tail to 11 amino acids decreased dramatically the signal of tail insertion and even more dramatically, the induction of the conformational change. In a substrate without tail (1 amino acid tail), the tail insertion and the conformational change were diminished to values similar to those found in control non ubiquitinated substrates. In the other hand, the degradation time was shortened in unstable substrates, as tested in substrate versions containing unstabilizing mutations in Titin-I27 (V13P or/and V15P) but identical tail lengths. However, containing supernumerary ubiquitin chains linked to the substrate did not influence degradation speed, in Titin-I27-based substrates including a long tail (35 amino acids). Thus, the presence of one, two or three ubiquitin chains did not impose any change in the speed of Titin-I27 degradation when carrying a competent initiation region.

Regarding the important aspect of the implications of polyubiquitin docking to substrate receptors, Bard et al. concluded that tetraubiquitin *per se* does not promote the transition from S1 to S3-like states. Therefore, the notion of ubiquitin as a protein attractor to proteasome, instead of a proteasomal activator, could be reinforced by these results, even though this important aspect would remain controversial. Peth and collaborators, from Goldberg lab, showed a sound correlation between ubiquitin conjugate binding and ATP hydrolysis, suggesting ATPase-activating properties of polyubiquitin [20]. The different methodological approaches of these works could explain the discrepancy on this important and still open aspect. Distinct protein conjugates used and the fact that Bard et al. worked with reconstituted proteasomes could explain it. Nonetheless, a key question is still whether ubiquitin could provide any spatial information to the proteasome that facilitates further processing steps, including downstream activation. A recent work from Cong and Glickman’s labs sheds light to this point [21]. In this work, the authors carried out cryo-EM analysis with yeast proteasomes incubated with ATP and K48-tetra ubiquitin. They observed a conformational change induced by the binding of tetra ubiquitin. Among the different conformational states that they observed, they found two sub-groups of “resting” proteasomes. One of them, C1-a, is assigned to the conventional S1 state. The other population, named C1-b proteasomes, was found to show a tilt of Rpn2, Rpn3, Rpn9, Rpn10 and Rpn12 subunits towards Rpn1, in a conformation that revealed higher stabilization of subunit movements. Similarly, they define C2-a and C2-b, C3-a and C3-b configurations, assigned to S2 and S3-4 previous established conformations. In this grouping nomenclature, “b” refers to ubiquitin bound proteasomes. In the case of C3-b, an additional shift in Rpn10 subunit and an extra density were observed, in an overall S4-like state. Analyzing in detail the mobility of subunits, they observe a higher score in Rpn1, Rpn2 and Rpn10 subunits in C1-a than in C1-b. They conclude that this decrease in subunit mobility is induced by K48-tetra ubiquitin, suggesting that ubiquitin-proteasome interaction could serve as a preparation for an activating conformation change (S1 to S2 transition). In the presence of tetraubiquitin, all the subunits involved in ubiquitin binding (Rpn1, Rpn2—by means of Rpn13—and Rpn10) come together, in a sort of closed conformation, that could facilitate further steps, such as substrate engagement and so on. Remarkably, the C1-b configuration was found to be dominant, representing the 43% of total particles in samples, showing the significance of this state. An additional very important observation made by Ding et al. corroborates data published in previous works [22]; they observed that Rpn1 exerts as a docking station for distinct important factors in substrate recruitment, in a non-competitive manner. Thus, they showed evidence of Rpn1-based alternative recruitment of Ubp6, Rad23 and tetraubiquitin.

## 3. Structure-Function Definition of Substrate Engagement, Deubiquitination and Translocation

Overall, initial steps in proteasome degradation include joint actions of ubiquitin, ubiquitin receptors, substrate initiation region, AAA+ motor entering pore and deubiquitinating subunits, to promote the correct engagement and transport of the substrate through the ATPase pore towards the CP. In that process, ATPase complex provides the mechanical energy to efficiently translocate the polypeptide chain while the protein is unfolded. The mechanism underlying this crucial step has been described by recent works from Martin and Mao labs. Remarkably, the principle of unfolding and translocation is the pulling force of the engine defined by AAA+ ATPases, as well described in these works. To approach that, De La Peña et al. [13] stalled protein translocation by inhibiting Rpn11 deubiquitinase activity with the inhibitor o-PA. It is important to point out that, proximal to rpn11, rpn10 exposes its ubiquitin interacting motif (UIM), playing a key role in appropriate ubiquitin recruitment and substrate positioning. Remarkably, Rpn10 is regulated by monoubiquitation, modification that restricts Rpn10 function by inhibiting the UIM and promoting Rpn10 dissociation [23,24,25]. Upon inhibition, they added to the proteasomes a protein with a single polyubiquitinated lysine adjacent to an unstructured C-terminal tail. The substrate was engaged by the flexible region, which penetrated the ATPase ring pore until the ubiquitin-lysine isopeptide bond residue established a contact with catalytically blocked Rpn11 active-site. The inhibition of Rpn11 acted as a trap for the substrate, which paralyzed the unstructured tail inside the ATPase-CP aligned tunnel. As argued by the authors, this wonderful structure is reminiscent of a proteasome in the act of reiterative pulling of a protein substrate partially unfolded due to thermodynamically stable domains which show unfolding resistance. In this situation, several fascinating and long-time unanswered aspects of proteasome mechanism are uncovered. The ubiquitin-lysine conjugate, containing the proximal ubiquitin molecule that represents the first link in the polyubiquitin chain, positions at the catalytic groove of Rpn11, which embraces the bond with an otherwise catalytically active β-hairpin. Descending into the CP, a straight narrow channel is defined by the ATPase hexameric motor, occupied by the stalled polypeptide in close proximity to a spiral staircase of tyrosine loops of ATPases (Rpt5 Y255, Rpt1 Y283, Rpt2 Y256, Rpt6 Y222 and Rpt3 Y246, from top to bottom) that circle or embrace the substrate. Importantly, the way that the ubiquitinated protein shows up in the complex is consistent with a mechanism of co-translocational deubiquitination in which the pulling forces are exerted by the ATPase AAA+ motor and Rpn11 acts as a sort of razor blade, removing ubiquitin protruding molecules with no additional motions.

In the Base-CP interphase, two distinct gating configurations are observed, with respect to CP alpha subunit N-termini. A common trait among Rpt2, Rpt3 and Rpt5 subunits is that their C-termini HbYX-motifs enter into the CP alpha ring intersubunit pockets, whereas the Rpt1 and Rpt6 C-terminal ends occupy the pockets in a variable manner. This renders the alpha CP subunits N-termini pointing upstream and making the internal proteolytic channel accessible to the translocating unfolded protein. Moreover, it is possible to corroborate that C-terminal ends of ATPases trigger the opening of the CP gate as previously described [26,27].

The choral action of the six ATPase subunits of the AAA+ motor has been described by several papers from different labs. In the mentioned work, De La Peña et al. showed that the conformations adopted by the ATPase subunits while they act on the translocation of the substrate are determined by distinct nucleotide states. They observe four distinct motor conformations showing nucleotide binding pockets occupied by different molecular densities. In these conformations, one or two ATPase subunits did not interact with the substrate, whereas the rest established contacts. The assignation of the status of the nucleotides inside the binding pockets during the progression of the hydrolysis cycle was carried out not only by density assessment but also by establishing the geometries of the ATPase sites, of the structural stability of the allosteric motifs and of the areas of contact between subunits. Interestingly, the subunits bound to ATP, competent for hydrolysis, formed a closed pocket with a larger area of contact, characterized by a direct interaction between the gamma phosphate of ATP and arginine side chains from the neighboring subunit. On the other hand, the subunits bound to ADP showed less intersubunit contacts and more flexible arginine residues, adopting a more open conformation. Subunits bound to ATP but not competent for hydrolysis, and those subunits in which hydrolysis just was catalyzed, showed very similar distances to Arg fingers; therefore, they were indistinguishable with this criterion. Thus, these pre- and post-hydrolysis sites were distinguished by assessments of pocket openness. It is observed a cyclic progression of nucleotide states synchronized with a wave of ATPase back and forward movements. That is, upon ATP hydrolysis and ADP production, the subunit shifts backwards creating the opening of the pore. In this shifted position, ADP is released and ATP is incorporated, and the ATP-bound subunit returns to its closed position. Thanks to the intersubunit communication provided by Arg fingers, this movement influences, and it is influenced by, neighboring subunits. In the direction of the hydrolyzing cycle, backwards shifts of ADP-bound subunits are promoted by inwards motions of preceding ATPases.

**Figure 3:**
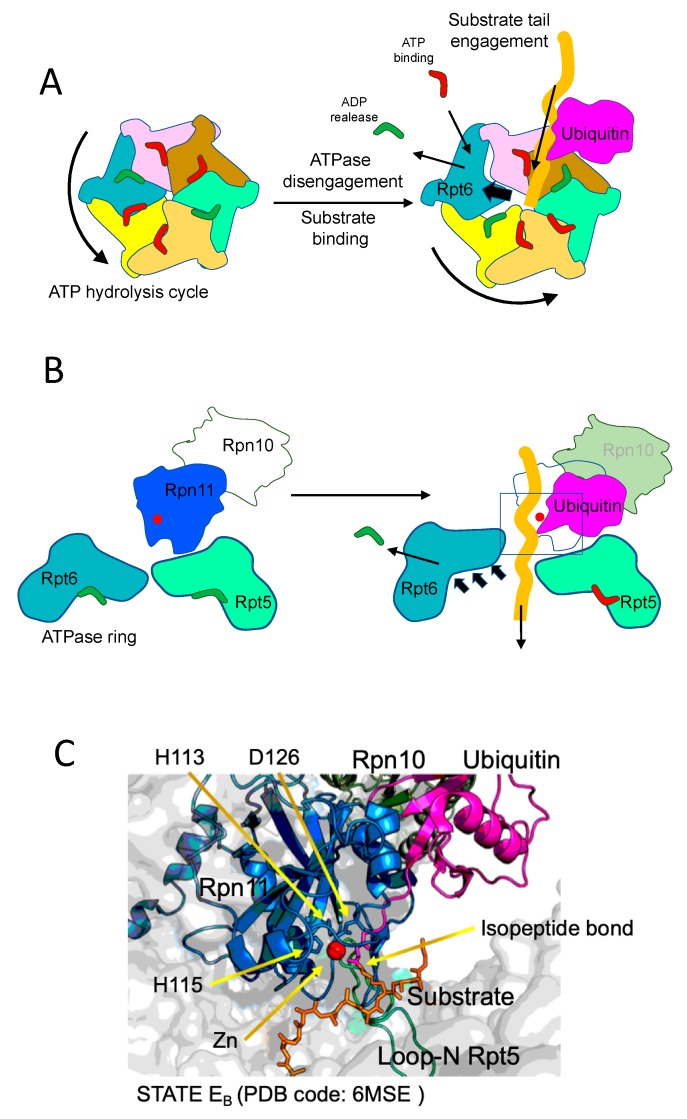
Representation of substrate engagement and deubiquitination state. (**A**) Top view of the ATPase ring similar to images provided by De La Peña et al. [13] and Dong et al. [27]. The transition from the inactive state (left) to the initial engagement of the substrate (right) is shown. ATP hydrolysis, ADP release and Rpt6 motion trigger the opening of the ATPase pore, facilitating the engagement of the tail of the substrate. (**B**) Side view of the process shown in A, with the representation of the movement back of Rpt6, generating additional space in the pore. A substrate in the process of engagement is included, simulating a formation of the E_B_ state. The rectangle included in the right image defines the space zoomed in the panel below. (**C**) Ribbon representation of the Rpn11 active site in the presence of a ubiquitinated substrate. Key components are included: catalytic Zinc (red), active-site Rpn11 (blue) residues (His113, His115 and Asp126), substrate (orange) and ubiquitin (pink) linked by means of an isopeptide bond, Rpn10 (dark green) and Rpt5 loop (light green). The pdb coordinates used to display this image in PyMOL: 6MSE (corresponding to E_B_ state, [27]). Abbreviations: ATP, adenosine triphosphate; ADP, adenosine diphosphate; Rpt, regulatory particle ATPase subunit; Rpn, regulatory particle non-ATPase subunit; pdb, protein data bank.

Importantly, these sequential states of nucleotide binding and release, and subunit motions, are coupled with substrate translocation. The concerted activity of each ATPase during substrate translocation generates several motor states in which subunits contact the substrate mostly when they are ATP-bound. In spiral movement, subunits push the polypeptide chain of the substrate, and the subunit most proximal to the CP gate hydrolyzes the ATP to form ADP, pyrophosphate is released and the ADP-bound subunit disengages the substrate, shifts backwards and up, then releases ADP, binds ATP and engages again the substrate in a new position. All subunits undergo this cycle in a sequential manner, and thus promote translocation (see Figure 3).

As mentioned above, an additional groundbreaking work has been recently published by the Youdong Mao Lab. In this work, an extensive cryo-EM analysis of proteasomes with an engaged ubiquitinated substrate is presented [28]. The work shows remarkable methodological differences with respect to De la Peña et al. [13], but outstanding coincidences as well. The major differences are that Dong et al. focused their work on human proteasomes, obtained from HEK293 cells, and the model substrate used was Sic1^PY^, a ubiquitinatable version of the Cdk1 inhibitor designed in previous works [29]. Another notable difference is that De la Peña et al. treated proteasomes with o-PA, in order to inhibit Rpn11 activity and maximize particles with trapped substrates in the process of translocation to the CP and containing intact ubiquitin-lysine isopeptide bond in the context of Rpn11 active site. Instead, Dong et al. performed a nucleotide substitution step by first priming proteasomes with ubiquitinated Sic1^PY^ and ATP, and afterwards supplying the system with slow-degradable ATPγS, in order to promote the binding of ATPγS and in this way chase proteasome particles at multiple different states, thus maximizing the heterogeneity of proteasomal states. This approach was successful because they observed and characterized up to seven distinct conformational states, covering initial substrate recognition (states E_A1_ and E_A2_, equivalent to S1), deubiquitinating state (E_B_, equivalent to S2), initial translocation states (E_C1_ and E_C2_, equivalent to S3) and active translocation/degradation conformations (E_D1_ and E_D2_, equivalent to S4). The comparative analysis of each state provided spatiotemporal information of the whole process.

Notably, they could define, in E_A_ states, a ubiquitin density in the context of Rpn1 T1 site, and two ubiquitin densities in the proximity of Rpt4-Rpt5 N-terminal coiled-coil (CC) domains and Rpn10, suggesting that a polyubiquitin entity can coordinate the simultaneous binding of multiple receptor surfaces during recruitment of the substrate to the ATPase pore. Moreover, a quaternary complex involving the substrate (ubiquitin-isopeptide bond-Sic1 moiety), Rpn11, Rpn8 and the N-loop of Rpt5 was observed in the E_B_ state. By comparing E_B_ state with precedent and posterior states they could describe the sequence of events that define substrate deubiquitination and its presentation to the AAA+ motor ATPases. The quaternary complex starts to form in the E_A2_ state, when substrate is still not engaged with the ATPase ring. From that state to the total engagement of the substrate, the authors describe a number of remarkable transitions. An important one is that Rpt4-Rpt5 CC domains shift up, shortening the distances of key groups of the quaternary complex. There is a progressive close up of proximal ubiquitin to Rpn11, and a distance of 3.5 Å between the isopeptide bond and the zinc atom of Rpn11 active site is reached, a distance fairly compatible with catalysis. Interestingly, the N-loop of Rpt5, which appears to be disordered in the E_A1_, E_C1,2_ and E_D1,2_ states, could have a specific role in E_B_ state, facilitating Rpn11-ubiquitin productive contacts and optimizing the orientation of the isopeptide bond. During the process, Rpn11 itself undergoes conformational changes in its insert-1 loop, which conforms one on the faces of the substrate binding pocket. The insert-1 loop is open in the E_A1_ state, it conforms a β-hairpin in the ubiquitin-bound states, as defined by De La Peña et al., and finally adopts a small, tight loop, in E_C2_ and E_D1,2_ states. The interactions and transitions observed in the quaternary complex would explain why Rpn11 is much less active in uncomplete proteasomal forms.

Furthermore, the authors describe in great detail the ATP and ADP bound states of the ATPase ring during substrate translocation. Notably, both Dong et al. and De La Peña et al. drew similar conclusions with respect nucleotide cycle and the principles of substrate translocation. Both works define a strong mechanistic coupling of activation conformational transitions with substrate engagement and deubiquitination; however, since Dong et al. captured higher diversity of conformational states, they were able to describe in more detail the structure-function basis of the transitions. ATP hydrolysis is controlling the whole process, from substrate engagement to substrate total translocation. In the E_A_ states the AAA+ motor channel is too narrow to engage a substrate. In the E_A_ to E_B_ transition, ATP hydrolysis and ADP release in Rpt6 triggers an iris-like movement in the whole ring that opens the axial channel. A major rotation of the Rpt6 AAA subdomain is observed, which creates the required space in the channel, followed by ADP release from Rpt6. This movement is accompanied by coordinated hydrolysis of other ATPase subunits, which increase the flexibility of the channel. In these conditions, substrate engagement takes place, and it is followed by the initiation of translocation. As also described by De La Peña et al., and detailed above, the process of translocation is promoted by sequential cycles of ATP binding, hydrolysis and ADP release, which trigger circular movements of the ATPase subunits. This process, which is ubiquitin-independent, creates the conditions, as translocation takes place, for the productive encounter of the substrate-ubiquitin isopeptide bond with the active site of Rpn11, thus facilitating the catalysis of deubiquitination (Figure 3).

Overall, due to the high level of conservation among different AAA+ motors, it is possible that the mechanochemical sequence of events defined in proteasomal ATPases may apply to other ATPase machines in nature, such as several unfoldase, disaggregase, extractase or cell cycle checkpoint remodeling complexes [30,31,32,33,34,35], uncovering a common fascinating solution found by evolution to reverse highly stable thermodynamic states of proteins. As a concluding remark, is should be highlighted that a notable level of proteasome structure/function mechanism characterization has been achieved. However, the upstream regulation of this sophisticated machine remains yet not understood. A complete movie of how upstream events control accessibility and degradability of substrates by the proteasome in the cell, together with how processivity is carried out in the proteasome to achieve total degradation of substrates, is still not available, although some of the scenes have been already recorded, as recent literature shows. In the present compilation, we have modestly selected, and commented in detail, some of the works that represent remarkable breakthroughs in the field, with no thoughtlessness towards other works that were not mentioned. Without a doubt, the full comprehension of protein degradation process will have a strong impact in biology and medicine, providing substantial basis for tackling important diseases. For example, in neurodegeneration, a future understanding and bioengineered control of activated-state proteasomes could open a new field of therapeutic approaches. Future efforts will be required to accomplish this fascinating challenge.
